# Machine Learning the Decoherence Property of Superconducting and Semiconductor Quantum Devices from Graph Connectivity

**DOI:** 10.3390/e28010089

**Published:** 2026-01-12

**Authors:** Quan Fu, Jie Liu, Xin Wang, Rui Xiong

**Affiliations:** 1 School of Physics and Technology, Wuhan University, Wuhan 430072, China; quanfu2-c@my.cityu.edu.hk; 2 Department of Physics, City University of Hong Kong, Tat Chee Avenue, Kowloon, Hong Kong SAR, China; jliu389-c@my.cityu.edu.hk (J.L.); x.wang@cityu.edu.hk (X.W.); 3 City University of Hong Kong Shenzhen Research Institute, Shenzhen 518057, China; 4 Quantum Science Center of Guangdong-Hong Kong-Macao Greater Bay Area, Shenzhen 518045, China

**Keywords:** supervised machine learning, superconducting qubit, semiconductor qubit, decoherence

## Abstract

Quantum computing faces significant challenges from decoherence and noise, which limit the practical implementation of quantum algorithms. While substantial progress has been made in improving individual qubit coherence times, the collective behavior of interconnected qubit systems remains incompletely understood. The connectivity architecture plays a crucial role in determining overall system susceptibility to environmental noise, yet systematic characterization of this relationship has been hindered by computational complexity. We develop a machine learning framework that bridges graph features with quantum device characterization to predict decoherence lifetime directly from connectivity patterns. By representing quantum architectures as connected graphs and using 14 topological features as input to supervised learning models, we achieve accurate lifetime predictions with $R^{2}>0.96$ for both superconducting and semiconductor platforms. Our analysis reveals fundamentally distinct decoherence mechanisms: superconducting qubits show high sensitivity to global connectivity measures (betweenness centrality $\delta_{1}=0.484$, spectral entropy $\delta_{1}=0.480$), while semiconductor quantum dots exhibit exceptional sensitivity to system scale (node count $\delta_{2}=0.919$, importance = 1.860). The complete failure of cross-platform model transfer ($R^{2}$ scores of −0.39 and −433.60) emphasizes the platform-specific nature of optimal connectivity design. Our approach enables rapid assessment of quantum architectures without expensive simulations, providing practical guidance for noise-optimized quantum processor design.

## 1. Introduction

Quantum computing has emerged as a transformative technology with the potential to solve classically intractable problems in optimization, simulation, and cryptography [1,2,3,4,5,6]. However, the practical realization of quantum algorithms faces significant challenges from decoherence and environmental noise, which cause quantum states to lose coherence and computational information to degrade [7,8,9]. While substantial research efforts have focused on improving individual qubit coherence times through material engineering and error correction techniques, the collective behavior of interconnected multiqubit systems remains less understood [10,11,12,13,14,15]. The connectivity architecture—the specific pattern of interqubit couplings—plays a crucial role in determining how noise propagates through quantum processors and ultimately limits computational fidelity [16,17].

The physical intuition that connectivity influences decoherence is supported by prior studies. For instance, Refs. [16,17] demonstrated that decoherence dynamics in semiconductor quantum dot systems are affected by their specific connectivity patterns, such as chains and rings. This establishes connectivity as a non-negligible factor. However, these works primarily analyzed a limited set of predefined geometries within a single hardware platform, leaving the relationship between connectivity and coherence times neither systematically quantified nor generalized across platforms.

Current approaches to characterizing noise properties in quantum processors rely heavily on numerical simulations of quantum dynamics or direct experimental measurements for each specific connectivity [17]. As qubit counts increase toward practical quantum advantage, the number of possible connectivity configurations grows combinatorially [18,19]. For *n* unlabeled nodes, the number of simple unweighted undirected connected graphs follows the sequence 2 (3 nodes), 6 (4), 21 (5), 112 (6), 853 (7), 11,117 (8), 261,080 (9). This extremely fast growth makes exhaustive characterization computationally prohibitive, creating an urgent need for efficient predictive models that can guide quantum processor design without requiring expensive simulations for every candidate connectivity.

The relationship between connectivity patterns and decoherence properties is particularly important because different quantum computing platforms exhibit distinct noise mechanisms that interact differently with architectural topology [20,21,22,23,24,25,26]. Superconducting qubits, which utilize macroscopic quantum states coupled through electromagnetic fields, experience noise propagation through photon-mediated interactions that can traverse the entire processor [27,28,29,30,31,32,33,34,35]. Semiconductor quantum dots, employing confined electrons with exchange interactions that decay rapidly with distance, exhibit more localized noise behavior dominated by charge fluctuations and nuclear spin effects [36,37,38,39,40,41,42,43]. Understanding how these platform-specific noise mechanisms interact with connectivity patterns is essential for optimizing quantum processor design.

We acknowledge that decoherence is a complex phenomenon dominated by material properties, specific noise spectra, and control imperfections. The premise of this work is not that decoherence is primarily a graph property, but that the connectivity layout is one identifiable and designable factor among many. Our goal is to isolate and quantify the predictive signal attributable to this structural aspect alone, providing a tool for rapid architectural screening within a broader, multi-faceted design optimization process.

In this work, we develop a comprehensive machine learning framework that bridges graph theory with quantum device characterization to address this challenge [44,45,46,47,48]. Building on the physical intuition that connectivity modulates noise propagation, we move beyond analyzing specific geometries. Our core approach is to systematically quantify connectivity through a comprehensive set of graph features. This allows for a scalable and comparative analysis of diverse layouts. We then explicitly test the applicability and generality of this structure-decoherence relationship across fundamentally different hardware paradigms, namely superconducting qubits and semiconductor quantum dots. Our main contributions include: a methodology for quantifying quantum architecture connectivity using graph topological features [49,50,51]; comprehensive datasets mapping connectivity patterns to decoherence lifetime for both major quantum computing platforms; comparative analysis of supervised learning models for predicting noise resilience from connectivity features; and identification of fundamental differences in how topological features influence decoherence across different physical implementations. The resulting framework provides a rapid assessment tool for quantum architectures, offering preliminary guidance on structural design choices that correlate with improved noise resilience.

The structure of this paper is organized as follows: Section 2 outlines the theoretical framework and methodology, covering the graph representation of connectivity patterns and the definition of topological features, followed by the decoherence metric and platform-specific noise models. Section 3 details the machine learning workflow, including dataset construction, model training, and evaluation protocols. Section 4 presents the results and analysis: it begins with a performance comparison of supervised learning models, examines the platform-specific correlation patterns and sensitivity differences, analyzes the rankings of feature importance, evaluates cross-platform generalization failure, and concludes with derived design implications. Finally, Section 5 synthesizes the key findings, discusses their physical interpretations and broader implications for quantum processor design, and suggests directions for future research.

## 2. Theoretical Framework and Methodology

### 2.1. Graph Representation and Topological Feature Set

We model quantum processor connectivity as undirected unweighted connected graphs G=(V,E), where nodes $V=\{v_{1},v_{2},\ldots ,v_{n}\}$ represent physical qubits and edges E⊆V×V represent implementable two-qubit gates. The connectivity graph defines both the available quantum operations and the pathways for noise propagation through the system.

**Figure 1:**
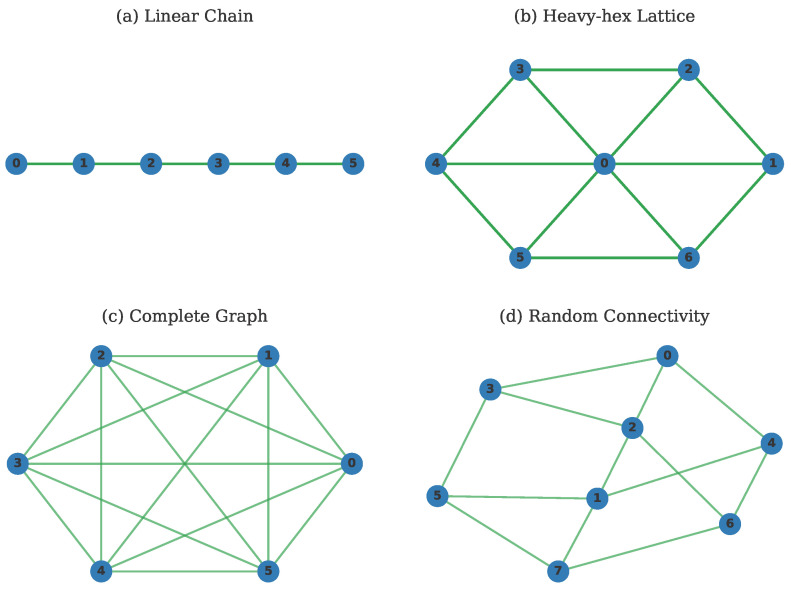
Representative connectivity graphs for quantum processors: (**a**) Linear chain, (**b**) Heavy-hex lattice, (**c**) Complete graph, and (**d**) Random connectivity pattern. Each node represents a physical qubit, and edges represent implementable two-qubit gates. The integer labels assigned to the nodes are used for identification in the graph model.

Figure 1 illustrates four representative connectivity patterns studied in this work, showcasing the diversity of quantum processor architectures. To systematically characterize these connectivity patterns, we compute 14 mathematical features for each graph, categorized into 6 groups based on their mathematical properties and physical interpretations, as summarized in Table 1 [50].

The basic structural features include the number of nodes n=|V| and edges m=|E|, which define the graph’s node set cardinality and connectivity density, determining graph density ρ=2m/n(n−1)∈[0,1] where ρ=1 for complete graphs and ρ=1/n for trees.

Distance-based connectivity metrics include the diameter $D=\max_{u\neq v}d\left(u,v\right)$, which quantifies worst-case communication cost in hops, and the average shortest path length $L=\frac{2}{n(n-1)}\sum_{u<v}d\left(u,v\right)$ serving as a global compactness measure. These metrics characterize the efficiency of information transfer through the network.

Algebraic connectivity $\lambda_{2}$, defined as the second smallest eigenvalue of the graph Laplacian L=D−A, is computed through the Rayleigh quotient minimization $\lambda_{2}=\min_{x⊥1}\frac{x^{T}Lx}{x^{T}x}$. This key resilience metric equals zero if and only if the graph is disconnected, with higher values indicating stronger global connectivity.

Local structural properties are captured by the average clustering coefficient $C=\frac{1}{n}\sum_{i=1}^{n}c_{i}$ where $c_{i}=2e_{i}/\left(k_{i}\left(k_{i}-1\right)\right)$ for $k_{i}\geq 2$ (zero otherwise), with $k_{i}$ denoting the number of edges incident to node *i* and $e_{i}$ representing the number of edges connecting pairs of neighbors of node *i*. This measure quantifies the tendency of nodes to form tightly connected groups, reflecting the modularity of the network structure.

Special graph properties include Eulerian circuit existence, determined by the condition that all nodes have even degrees in a connected graph, and planarity tested via Kuratowski’s theorem [52]. These properties have implications for the physical realizability and routing efficiency of the corresponding quantum processor.

Degree-related statistics encompass the mean ${µ}_{k}=\frac{1}{n}\sum k_{i}$, standard deviation $\sigma_{k}=\sqrt{\frac{1}{n}\sum {\left(k_{i}-{µ}_{k}\right)}^{2}}$, and skewness $\gamma_{k}=\frac{n\sum {\left(k_{i}-{µ}_{k}\right)}^{3}}{\left(n-1\right)\left(n-2\right)\sigma_{k}^{3}}$ of the degree distribution. The assortativity coefficient *r* measures the correlation between degrees of adjacent nodes.

Centrality and spectral features include the mean betweenness centrality $\bar{b}=\frac{1}{n}\sum_{i}b_{i}$ where $b_{i}=\sum_{s\neq i\neq t}\sigma_{st}\left(i\right)/\sigma_{st}$ identifies critical bottleneck nodes; here $\sigma_{st}$ is the total number of shortest paths from node *s* to node *t*, and $\sigma_{st}\left(i\right)$ is the number of those paths that pass through node *i*. The spectral entropy $S=-\sum \left(\lambda_{i}/\sum \lambda_{j}\right)\ln\left(\lambda_{i}/\sum \lambda_{j}\right)$ is calculated from the Laplacian eigenvalue distribution $\left\{\lambda_{i}\right\}$, measuring the structural complexity of the graph.

The noise sensitivity metric $\eta =\frac{1}{n}\sum k_{i}^{2}$ emphasizes the influence of high-degree nodes on error propagation, providing a second-moment measure of connectivity heterogeneity. This comprehensive feature set enables multidimensional characterization of graph topology relevant to quantum noise resilience.

The relative importance of these graph features, as identified by our models, offers physical insights into how connectivity influences decoherence. A high algebraic connectivity suggests a globally robust network where noise or excitation may diffuse more rapidly, potentially preventing localized error buildup. The clustering coefficient quantifies local connectivity triangles; a high value may indicate regions prone to crosstalk or collective dynamics, directly impacting localized decoherence processes. Statistics of the degree distribution (mean, deviation, skewness) describe connectivity heterogeneity; for instance, high positive skewness implies hub nodes exist, which could act as central points for error propagation. Assortativity reveals whether highly connected nodes link to each other, influencing correlated error patterns. Betweenness centrality identifies bottleneck qubits in information paths, which may be critical failure points under noise. Spectral entropy reflects the diversity of structural modes in the system, which may couple differently to environmental noise spectra. The noise sensitivity metric, emphasizing high degree nodes, directly targets the network’s vulnerability to perturbations at hubs. Finally, planarity is a fundamental hardware feasibility constraint. Its importance in prediction would indicate that the physical requirement of a 2D layout substantially limits the achievable coherence resilient graphs. Conversely, low importance suggests that optimal graphs for coherence are often compatible with planar fabrication, simplifying experimental design. Thus, these features collectively map abstract graph properties to tangible physical concepts like error diffusion, crosstalk, bottleneck vulnerability, and structural feasibility, bridging the gap between topological characterization and quantum hardware performance.

### 2.2. Decoherence Metric and Noise Models

We quantify noise resilience through quantum state preservation fidelity under identity evolution. For each connectivity graph *G*, we simulate evolution under the identity operation with platform-specific noise models and compute the fidelity decay $F\left(t\right)=\langle \psi_{0}|U^{-iH_{\mathrm{noise}}t}|\psi_{0}\rangle$, where $|\psi_{0}\rangle =|↑↑\ldots \rangle$ and $H_{\mathrm{noise}}$ is determined later, then extract the characteristic lifetime τ via exponential fitting $F\left(t\right)∼e^{-t/\tau }$. The lifetime τ serves as our primary metric for noise resilience, with larger values indicating better coherence properties. The exponential decay constant τ is extracted through least-squares fitting to the fidelity curve over the simulation timeframe, providing a robust measure of the coherence time under each connectivity configuration.

For superconducting qubit systems, the noise Hamiltonian incorporates the dominant decoherence mechanisms observed in transmon and fluxonium architectures:

(1) $$H_{\mathrm{sc}}=\sum_{i}\delta \omega_{i}\sigma_{z}^{i}+\sum_{\langle i,j\rangle }J_{zz}^{ij}\sigma_{z}^{i}\sigma_{z}^{j}+\sum_{i}\Gamma_{i}\sigma_{+}^{i}\sigma_{-}^{i}+\sum_{i}\Gamma_{\phi }^{i}\sigma_{z}^{i},$$

where $\delta \omega_{i}$ represents single-qubit frequency fluctuations due to 1/f charge noise and flux noise, $J_{zz}^{ij}$ denotes static ZZ coupling variations arising from capacitive and inductive crosstalk, $\Gamma_{i}$ captures energy relaxation ($T_{1}$ processes), and $\Gamma_{\phi }^{i}$ represents pure dephasing from high-frequency noise components. The noise parameters are normalized to the characteristic coupling strength $g_{0}\approx 2\pi \times 10$ MHz with typical ranges: $\delta \omega_{i}/g_{0}$ = 0.5–5% for frequency fluctuations (corresponding to 50–500 kHz), $J_{zz}^{ij}/g_{0}$ = 0.5–5% for coupling variations, $\Gamma_{i}/g_{0}$ = 0.1–1% for relaxation rates (corresponding to $T_{1}=1/\Gamma_{i}\approx$ 1–10 µs), and $\Gamma_{\phi }^{i}/g_{0}$ = 0.2–2% for dephasing rates. These ranges reflect experimental observations in state-of-the-art transmon processors with coherence times $T_{2}^{*}\approx$ 1–100 µs.

For semiconductor quantum dot systems, we model the dominant noise sources consistent with GaAs and Si/SiGe implementations:

(2) $$\begin{array}{c} H_{\mathrm{semi}}=\sum_{i}\left(\Delta_{\mathrm{charge}}^{i}+\Delta_{\mathrm{nuclear}}^{i}\right)\sigma_{z}^{i}+\sum_{\langle i,j\rangle }\delta J_{H}^{ij}\left(\sigma_{x}^{i}\sigma_{x}^{j}+\sigma_{y}^{i}\sigma_{y}^{j}+\sigma_{z}^{i}\sigma_{z}^{j}\right)+\sum_{i}\Gamma_{\mathrm{charge}}^{i}\sigma_{z}^{i} \end{array}$$

where $\Delta_{\mathrm{charge}}^{i}$ represents charge noise-induced detuning fluctuations with 1/f spectral density, $\Delta_{\mathrm{nuclear}}^{i}$ captures Overhauser field fluctuations from nuclear spins, $\delta J_{H}^{ij}$ denotes exchange coupling variations due to charge noise, and $\Gamma_{\mathrm{charge}}^{i}$ accounts for charge noise-induced dephasing. The parameters are normalized to the Heisenberg exchange coupling $J_{H}\approx 2\pi \times 1$ MHz with typical ranges: $\Delta_{\mathrm{charge}}^{i}/J_{H}$ = 5–20% for charge noise (dominant in GaAs), $\Delta_{\mathrm{nuclear}}^{i}/J_{H}$ = 1–5% for nuclear noise (more significant in GaAs than Si), $\delta J_{H}^{ij}/J_{H}$ = 2–10% for coupling variations, and $\Gamma_{\mathrm{charge}}^{i}/J_{H}$ = 3–15% for charge noise-induced dephasing. These parameters correspond to typical coherence times $T_{2}^{*}\approx$ 10–100 ns in semiconductor quantum dots, with charge noise being the primary limitation in current devices.

To faithfully represent the statistical variability and spectral properties of noise in experimental quantum devices, we implement platform specific noise models using a combined approach of probabilistic sampling and time series generation. For superconducting qubits, we model frequency and coupling noise with Gaussian distributions (standard deviations of 2% and 3%, respectively), while relaxation and dephasing rates follow exponential distributions. For semiconductor quantum dots, charge noise parameters are modeled with log-normal distributions to capture 1/f spectral characteristics, and nuclear noise with Gaussian distributions. We account for both quasi-static and dynamic noise components. For quasi-static noise where fluctuations occur on timescales much longer than typical gate operations, we employ a Monte Carlo method: parameters such as coupling strengths $J_{zz}^{ij}$ are sampled from Gaussian distributions $J_{zz}^{ij}∼N\left(J_{zz,0}^{ij},\sigma^{2}\right)$ and held constant over each stochastic realization. For dynamic 1/f noise, we generate time dependent fluctuations (e.g., in local frequencies $\delta \omega_{i}\left(t\right)$) using the fractional Brownian motion (fBm) method to accurately reproduce the characteristic power spectral density [53]. This hybrid approach, combining Gaussian based Monte Carlo sampling for quasi-static noise and fBm generated time series for 1/f noise, ensures that our simulations capture both the statistical variability and the temporal spectral properties observed in real superconducting and semiconductor quantum devices.

## 3. Machine Learning Methodology

A key methodological choice in this study is the exclusive use of graph features as model inputs to predict the simulated decoherence times. This approach is designed to test the upper bound of predictive information that can be extracted from connectivity structure alone, isolating its effect from other dominant physical factors. It is important to note that we do not treat lifetime as purely structural properties; rather, we model the component of their variability that can be attributed to differences in layout, acknowledging that the absolute values are determined by a complex interplay of many physical processes.

**Figure 2:**
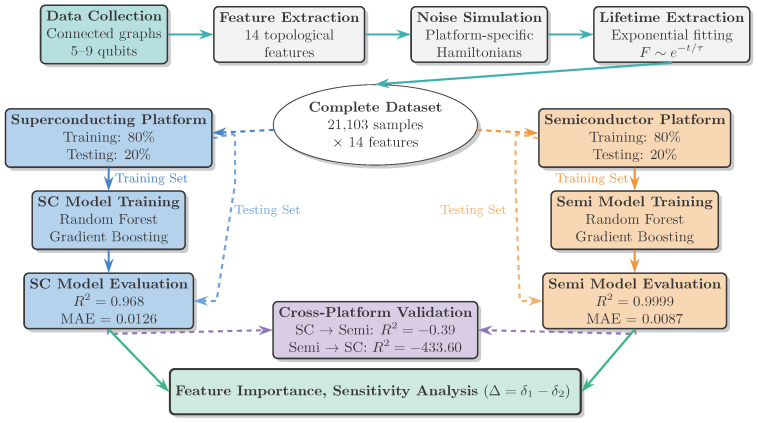
Comprehensive workflow of the machine learning framework: From graph generation to lifetime prediction, including feature extraction, noise simulation, model training, and cross-platform validation.

Figure 2 illustrates the comprehensive workflow of our machine learning framework. We generate the complete set of connected graphs for 5–8 qubits using established algorithms, resulting in 11,103 unique graph configurations (5 nodes: 21 graphs, 6 nodes: 112 graphs, 7 nodes: 853 graphs, 8 nodes: 11,117 graphs). For 9-qubit systems, we sample 10,000 representative connected graphs from the total 261,080 possible configurations to maintain computational tractability while ensuring adequate coverage of the design space. Our dataset comprises 10,000 unique (non-isomorphic) connected graphs with 9 nodes. To construct it, we uniformly sampled from the space of connected graphs by repeatedly generating candidate graphs using the Erdős–Rényi model and retaining only connected ones. We then performed an isomorphism check to ensure all graphs in the final dataset are distinct. For each quantum computing platform, we construct separate datasets with platform-specific noise characteristics. Our study employs a graph abstraction to represent qubit connectivity. While this provides a powerful framework for comparative analysis, it is necessarily a simplification. For semiconductor quantum dots in particular, the abstraction of an edge as a theoretically allowed coupling does not capture the full complexity of experimental fabrication, control, and scalability constraints. The feasibility and fidelity of specific couplings depend on details like gate design, material disorder, and pulse sequences, which are beyond the scope of this graph model. Therefore, our work should be viewed as a first level structural screening tool within a larger hardware co-design pipeline. It identifies promising connectivity archetypes from a vast combinatorial space, which must subsequently be validated and refined against detailed physical models and experimental constraints. The strong predictive power of simple graph features even within this idealized framework suggests that connectivity topology is a significant, separable factor worthy of such focused optimization.

The dataset construction pipeline follows a consistent four-step process for each connectivity pattern: construct quantum processor model with platform-appropriate Hamiltonian; simulate identity gate evolution with calibrated noise parameters; compute fidelity decay curves and extract characteristic lifetime τ; compute all 14 graph topological features. The final datasets contain feature matrices $X_{\mathrm{sc}}\in R^{21,103\times 14}$ and $X_{\mathrm{semi}}\in R^{21,103\times 14}$ with corresponding target vectors $y_{\mathrm{sc}},y_{\mathrm{semi}}\in R^{21,103}$.

We implement a dual-platform supervised learning approach with platform-specific model training [54,55,56,57,58,59,60,61]. The data preprocessing includes feature standardization through z-score normalization, 80–20% train-test split with stratified sampling, and 5-fold cross-validation (CV) for robust hyperparameter optimization. The model architecture selection focuses on Random Forest Regressor and Histogram-based Gradient Boosting, with platform-optimized hyperparameters. These relatively simple, interpretable models were chosen for this initial exploratory study for two primary reasons. First, they provide a robust baseline and allow for direct feature importance analysis, which is crucial for gaining physical insights into which graph properties most influence decoherence. Second, given the moderate size of our dataset, these models mitigate the risk of overfitting and ensure generalizability. We acknowledge that more advanced architectures such as Graph Convolutional Networks (GCNs)—which operate directly on graph adjacency matrices and could capture more nuanced structural representations—or uncertainty-quantification methods (e.g., dropout-based Bayesian neural networks) represent promising future directions. Such methods would be particularly valuable as the library of quantum device layouts expands or when predicting more granular properties (e.g., per-qubit coherence times or full noise spectra). However, for the present goal of establishing a interpretable relationship between graph features and decoherence times, the chosen models provide a suitable and transparent starting point.

The evaluation framework employs two complementary metrics: Mean Absolute Error (MAE) and the Coefficient of Determination ($R^{2}$) [54,55,56]. We also employ permutation importance analysis with platform-specific validation to identify the most influential graph features for predicting decoherence properties.

## 4. Results and Analysis

### 4.1. Supervised Learning Performance Comparison

The complete dataset is initially split into 80% for training and 20% for testing. Model hyperparameters are tuned via 5-fold cross-validation on the training set using Randomized Search CV (from scikit-learn) [62]. Once optimal hyperparameters are identified, the Randomized Search CV automatically refits the best model on the entire training set. The final performance metrics and all subsequent analyses (feature importance, etc.) are reported based on the evaluation of this final model on the held-out test set. The supervised learning results demonstrate exceptional prediction accuracy for both quantum computing platforms, as summarized in Table 2. Semiconductor qubits achieve near-perfect predictability ($R^{2}=0.9999$) with both Histogram Gradient Boosting and Random Forest models, while superconducting qubits show slightly lower but still outstanding performance ($R^{2}=0.9682$). This performance differential reflects the different relationships between connectivity patterns and decoherence mechanisms in the two platforms.

The MAE values of 0.0126 for superconducting and 0.0087 for semiconductor platforms correspond to approximately 1% relative error in lifetime prediction, representing exceptional accuracy for quantum device characterization. The consistency between both algorithms across platforms indicates robust learned relationships that are not algorithm-dependent.

**Figure 3:**
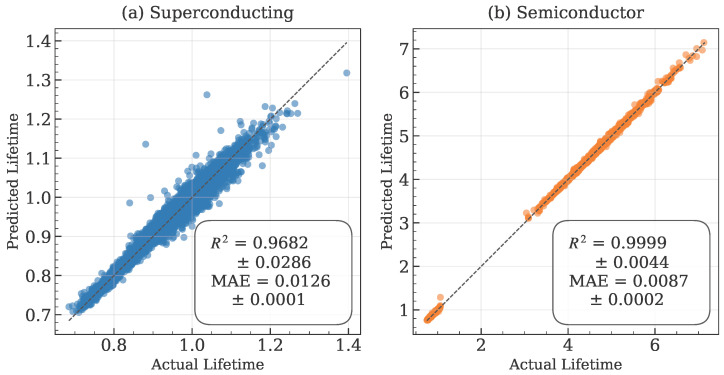
Predicted vs. actual lifetime values for (**a**) superconducting and (**b**) semiconductor platforms using the best-performing model, which is Histogram Gradient Boosting. The near-perfect alignment for semiconductor platforms reflects the deterministic relationship between node count and coherence time, as shown in Section 4.2 and Section 4.3. Values are reported as mean standard deviation from 5-fold cross-validation.

The agreement between predicted and actual lifetime values, displayed in Figure 3, further validates the model performance. Semiconductor platforms show almost perfect alignment with the ideal prediction line, while superconducting platforms exhibit slight dispersion that reflects the more complex relationship between multiple topological features and decoherence in these systems.

### 4.2. Platform-Specific Correlation Analysis and Sensitivity Differences

To understand the underlying mechanisms driving the supervised learning performance, we analyze the feature-lifetime correlations and their platform-specific differences. The correlation coefficients (denoted as $\delta_{s}$) quantify the strength and direction of the relationship between each topological feature and the extracted lifetime, computed using Spearman’s rank correlation method, which assesses monotonic relationships between variables [63]. For two variables *X* and *Y* with *l* observations, Spearman’s correlation is calculated as follows:

(3) $$\delta_{s}=1-\frac{6\sum d_{i}^{2}}{l(l^{2}-1)},$$

where $d_{i}$ is the difference between the ranks of corresponding variables, s=1,2 for superconducting and semiconductor platforms respectively. This non-parametric measure is particularly suitable for our analysis as it does not assume linearity and is robust to outliers, making it ideal for capturing the complex relationships between graph topological features and quantum coherence properties. The platform sensitivity difference metric Δ is calculated as $\Delta =\delta_{1}-\delta_{2}$, where positive values indicate features more predictive in superconducting platforms and negative values highlight semiconductor-sensitive features.

**Figure 4:**
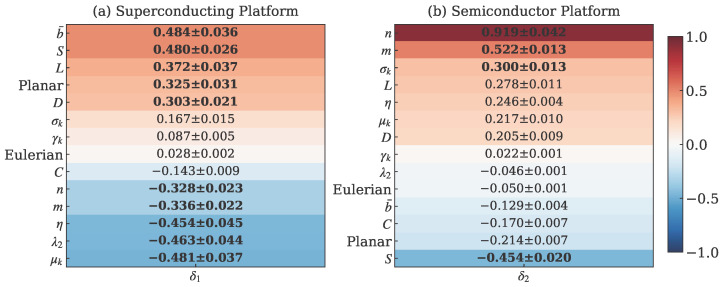
Feature-lifetime correlation matrices: (**a**) Superconducting platform showing strong positive correlation with centrality measures ($\bar{b}$: $\delta_{1}=0.484$, *S*: $\delta_{1}=0.480$), (**b**) Semiconductor platform with exceptionally strong correlation with node count (*n*: $\delta_{2}=0.919$). Values in each cell are reported as correlation coefficient ± standard deviation from 5-fold cross-validation.

Figure 4 presents the feature-lifetime correlation matrices for both quantum computing platforms. For superconducting qubits, global connectivity measures are the primary determinants of noise resilience. Betweenness centrality $\bar{b}$ achieves the highest correlation coefficient $\delta_{1}=0.484$, closely followed by spectral entropy *S* with $\delta_{1}=0.480$. This pattern indicates that information flow efficiency and structural complexity significantly influence coherence times in superconducting systems. Distance-based measures including average shortest path length (*L*: $\delta_{1}=0.372$) and diameter (*D*: $\delta_{1}=0.303$) also show substantial positive correlations, reinforcing the importance of compact architectures that minimize communication distances.

In contrast, semiconductor quantum dots exhibit a completely different correlation pattern dominated by system scale rather than connectivity efficiency. Node count (*n*) demonstrates an exceptionally strong positive correlation ($\delta_{2}=0.919$), indicating that larger systems achieve superior noise resilience in these implementations. Edge count (*m*: $\delta_{2}=0.522$) and degree statistics ($\sigma_{k}$: $\delta_{2}=0.300$) also contribute significantly, while spectral features show weak or negative correlations. This contrast highlights the platform-specific nature of optimal connectivity design, with superconducting systems benefiting from efficient global connectivity and semiconductor systems thriving on scalable architectures.

The correlation structure further reveals that negative relationships characterize certain features in each platform. For superconducting qubits, node count (*n*: $\delta_{1}=-0.328$), edge count (*m*: $\delta_{1}=-0.336$), and algebraic connectivity ($\lambda_{2}$: $\delta_{1}=-0.463$) show inverse relationships with coherence time, suggesting potential challenges with noise propagation in larger, more connected architectures. Semiconductor platforms exhibit negative correlations with spectral entropy (*S*: $\delta_{2}=-0.454$) and planarity (Planar: $\delta_{2}=-0.214$), indicating that global structural properties may be less relevant for systems dominated by localized noise mechanisms.

**Figure 5:**
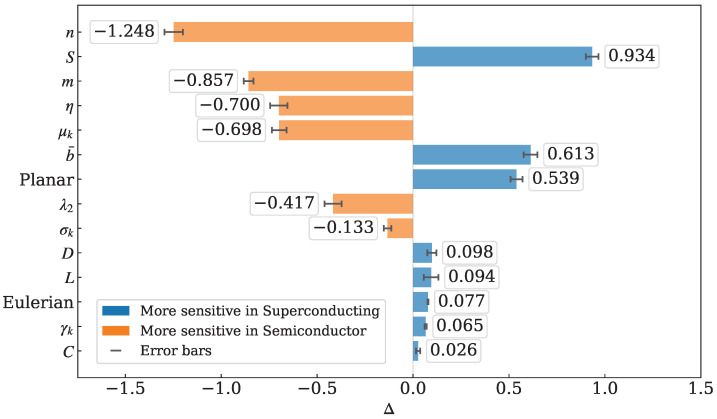
Platform sensitivity differences: Bar plot showing correlation differences ($\Delta =\delta_{1}-\delta_{2}$) for key graph features. Positive Δ values indicate greater sensitivity in superconducting platforms, negative values indicate greater sensitivity in semiconductor platforms. Error bars represent standard deviations from 5-fold cross-validation. In some cases, error bars are not visible due to their small magnitude relative to the bar length.

Figure 5 presents the platform sensitivity differences quantified through the Δ metric. The sensitivity difference analysis reveals clear technological specialization. Features with strong superconducting preference (Δ>0.5) include spectral entropy (Δ=+0.934), betweenness centrality (Δ=+0.613), and planarity (Δ=+0.539), representing global structural properties that influence electromagnetic noise propagation. These features show substantially stronger correlations with coherence time in superconducting systems compared to semiconductor platforms, confirming that superconducting qubits benefit from architectures that optimize information flow efficiency and global connectivity.

Conversely, features with semiconductor preference (Δ<−0.5) include node count (Δ=−1.248), edge count (Δ=−1.008), and noise sensitivity (Δ=−0.700), reflecting the importance of scalable architectures and localized noise containment in semiconductor quantum dots. The negative Δ values signify that these features have stronger correlations with coherence time in semiconductor platforms, highlighting the dominance of system scale over topological complexity in these implementations.

The sensitivity analysis further reveals that certain features, including clustering coefficient (*C*: Δ=+0.026) and degree skewness ($\gamma_{k}$: Δ=+0.065), show minimal platform differentiation with Δ values close to zero. These near-zero differentials indicate that the correlation strengths for these features are approximately equal across both platforms, suggesting they may represent more universal aspects of quantum processor design that translate across different physical implementations.

The combined analysis of correlation patterns and sensitivity differences provides a comprehensive understanding of the platform-specific optimization requirements for quantum processor design. The clear differentiation in feature sensitivity, as visualized in both Figure 4 and Figure 5, underscores the necessity for distinct design methodologies that account for the unique physical mechanisms governing decoherence in each quantum computing technology.

### 4.3. Feature Importance Rankings and Platform Specificity

**Figure 6:**
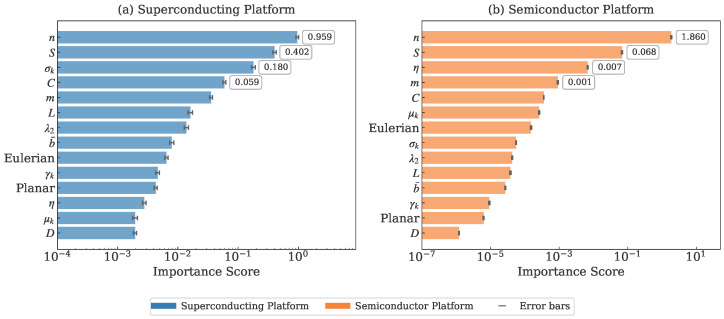
Feature importance rankings from permutation importance analysis: (**a**) Superconducting platform showing distributed importance across multiple features, (**b**) Semiconductor platform with extreme dominance of node count (*n*: 1.860). Error bars represent standard deviations from 5-fold cross-validation. In some cases, error bars are not visible due to their small magnitude relative to the bar length.

The feature importance rankings derived from permutation importance analysis provide further insights into the platform-specific prediction mechanisms, as shown in Figure 6.

For superconducting qubits, the importance distribution shows a balanced contribution from multiple feature categories, with node count (*n*: 0.959), spectral entropy (*S*: 0.402), and degree standard deviation ($\sigma_{k}$: 0.180) all contributing significantly. This distributed pattern reflects the complex interplay between system size, structural complexity, and connectivity heterogeneity in determining superconducting qubit coherence.

In contrast, semiconductor qubits exhibit a dramatically concentrated importance structure dominated overwhelmingly by node count (*n*: 1.860), which is approximately 27 times more important than the second-ranked feature. This extreme concentration explains the near-perfect predictability observed in semiconductor platforms and underscores the primacy of system scale in these implementations.

### 4.4. Cross-Platform Generalization Analysis

The cross-platform transfer analysis reveals catastrophic failure in generalization between quantum computing technologies, as documented in Table 3. Models trained on one platform perform disastrously when applied to the other, with profoundly negative $R^{2}$ scores of −0.39 for superconducting-to-semiconductor transfer and −433.60 for the reverse direction.

This complete generalization failure underscores the fundamental incompatibility of decoherence mechanisms between the two platforms. The extreme negative transfer performance indicates that the feature-target relationships learned in one platform are not merely insufficient but actively destructive when applied to the other platform, highlighting the necessity for platform-specific design methodologies.

### 4.5. Design Implications and Optimization Guidelines

The comprehensive analysis of prediction performance, correlation patterns, and feature importance yields clear design implications for quantum processor architecture optimization. The platform specific nature of optimal connectivity design necessitates distinct approaches for each quantum computing platform. These hardware level insights complement recent advances in quantum protocol design, such as privacy preserving communication systems for smart grids based on semi-quantum computation [64], highlighting the multidimensional nature of quantum technology development where both hardware resilience and protocol security must be addressed.

For superconducting quantum processors, the analysis indicates that architectures should prioritize global connectivity properties to maximize noise resilience. The strong positive sensitivity differences for spectral entropy (Δ=+0.934), betweenness centrality (Δ=+0.613), and planarity (Δ=+0.539) demonstrate that these platforms benefit from enhanced structural complexity, efficient information flow pathways, and planar routing compatibility to reduce electromagnetic crosstalk. Rather than focusing on single-parameter optimization, superconducting systems achieve optimal performance through balanced integration of multiple topological features that collectively distribute noise propagation while maintaining computational connectivity.

Semiconductor quantum processors exhibit fundamentally different optimization requirements, with scalable architectures providing superior noise resilience. The strong negative sensitivity differences for node count (Δ=−1.248), edge count (Δ=−1.008), and degree statistics (Δ=−0.698) indicate that these platforms thrive on appropriate system scaling, controlled connectivity density, and regular degree distributions to mitigate localized charge noise effects. Modular design approaches that contain noise propagation within localized regions are particularly effective for semiconductor implementations, leveraging the deterministic relationship between system scale and coherence time while maintaining the interaction strength necessary for quantum operations.

The quantitative sensitivity profiles derived from our analysis provide a rigorous foundation for designing noise-resilient quantum processors. By tailoring architectural choices to the specific physical mechanisms governing decoherence in each platform, designers can maximize coherence times and overall system performance. The clear differentiation between platform requirements underscores that optimal connectivity patterns are not universally applicable but must be specifically engineered for each quantum computing technology based on its unique noise characteristics and operational constraints. This hardware centric approach to quantum system design represents a complementary perspective to protocol focused quantum research, together advancing the robustness and practicality of quantum technologies across computing and communication domains.

## 5. Discussion and Conclusions

We have developed and validated a comprehensive supervised machine learning framework for predicting quantum processor lifetime directly from connectivity graphs. Our analysis reveals fundamentally distinct relationships between topological features and noise resilience in superconducting and semiconductor quantum computing platforms.

For superconducting qubits, global connectivity measures—particularly betweenness centrality ($\delta_{1}=0.484$) and spectral entropy ($\delta_{1}=0.480$)—are the primary determinants of noise resilience, reflecting the electromagnetic nature of noise propagation in these systems. The supervised learning models achieve excellent prediction accuracy ($R^{2}=0.9682$) with distributed feature importance across multiple topological categories. Semiconductor quantum dots exhibit completely different behavior, with node count demonstrating both the strongest correlation ($\delta_{2}=0.919$) and feature importance (1.860), indicating that system scale is the dominant factor in these implementations. The near-perfect predictability ($R^{2}=0.9999$) reflects the deterministic relationship between connectivity and decoherence in semiconductor platforms.

The complete failure of cross-platform model transfer, with $R^{2}$ scores of −0.39 for superconducting-to-semiconductor and −433.60 for the reverse direction, underscores the fundamental incompatibility of decoherence mechanisms between the two platforms. This result demonstrates that connectivity optimization must be performed separately for each quantum computing platform, and that findings from one platform cannot be extrapolated to others.

We clarify that the graph adjacency matrix does not itself generate decoherence; rather, it defines the interaction pathways through which platform specific fundamental noise processes (e.g., dielectric loss, 1/f charge noise, crosstalk) couple into and propagate across the qubit network. In our simulations, the noise models are physically motivated and platform specific. The graph topology acts as a scaffold that shapes the collective dynamics arising from these underlying sources. For example, in superconducting circuits with global electromagnetic coupling, high betweenness centrality may pinpoint qubits that act as bottlenecks for photon mediated noise propagation. In semiconductor dots with localized exchange interactions, the node count may dominate as it scales the number of potential local noise channels. Thus, our machine learning model learns how structural connectivity systematically modulates the impact of fundamental physical noise on the observed decoherence times, transforming graph patterns from a numerical observation into a quantitative descriptor of architectural influence on noise resilience.

The computational efficiency of our approach enables rapid exploration of the combinatorial design space of quantum processor connectivity. This work establishes a proof of concept that structural connectivity, as quantified by graph theory, carries significant predictive information for decoherence times—a relationship that had been suggested but not systematically generalized across platforms. We emphasize the exploratory nature of this study. Our model successfully identifies structural correlates of coherence times, offering a valuable rapid screening tool for the early stages of quantum processor design. However, it represents a focused investigation into one factor among many. A complete physical model of decoherence must integrate material, control, and environmental factors alongside connectivity. The value of our framework lies in its ability to provide preliminary, data-driven guidance on architectural choices within a broader co-design pipeline, not in presenting a deterministic or exhaustive theory of decoherence.

Future research directions include extending the analysis to additional quantum computing platforms such as trapped ions and photonic qubits, incorporating dynamic connectivity patterns and weighted edges to better model physical systems, and developing multi-objective optimization approaches that balance coherence time with other performance metrics such as gate fidelity and connectivity constraints.

## Figures and Tables

**Table 1:** Summary and description of the graph features used as predictors in the machine learning models.

Feature Category	Specific Features	Mathematical Definition
Basic Structural	Number of nodes (*n*), edges (*m*), density (ρ)	n=|V|, m=|E|, density $\rho =\frac{2m}{n(n-1)}$
Distance-Based	Diameter (*D*), average shortest path (*L*)	D=maxd(u,v), $L=\frac{2}{n(n-1)}\sum d\left(u,v\right)$
Algebraic	Algebraic connectivity ($\lambda_{2}$)	Second smallest Laplacian eigenvalue: $\lambda_{2}\left(L\right)$
Local Structural	Clustering coefficient (*C*)	$C=\frac{1}{n}\sum c_{i}$, $c_{i}=\frac{2e_{i}}{k_{i}\left(k_{i}-1\right)}$ for $k_{i}\geq 2$
Special Properties	Eulerian, planarity	Eulerian: all even degrees; planar: no edge crossings
Degree Statistics	Mean (${µ}_{k}$), std ($\sigma_{k}$), skewness ($\gamma_{k}$)	Moments of degree distribution: ${µ}_{k}=\frac{1}{n}\sum k_{i}$
Centrality	Betweenness centrality ($\bar{b}$)	$b_{i}=\sum \frac{\sigma_{st}\left(i\right)}{\sigma_{st}}$, $\bar{b}=\frac{1}{n}\sum b_{i}$
Spectral	Spectral entropy (*S*)	$S=-\sum \left(\frac{\lambda_{i}}{\sum \lambda_{j}}\right)\ln\left(\frac{\lambda_{i}}{\sum \lambda_{j}}\right)$
Robustness	Noise sensitivity (η)	$\eta =\frac{1}{n}\sum k_{i}^{2}$

**Table 2:** Supervised learning performance comparison for both platforms. Values are reported as mean standard deviation from 5-fold cross-validation.

	Histogram Gradient Boosting	Random Forest
Superconducting		
$R^{2}$	0.9682 ± 0.0286	0.9651 ± 0.0187
MAE	0.0126 ± 0.0001	0.0131 ± 0.0001
Semiconductor		
$R^{2}$	0.9999 ± 0.0044	0.9999 ± 0.0026
MAE	0.0087 ± 0.0002	0.0091 ± 0.0001

**Table 3:** Cross-platform model transfer performance ($R^{2}$ scores). The arrow (→) indicates the training-to-testing direction: model trained on the left platform and tested on the right platform.

Model	Training → Testing	$R^{2}$ Score
Histogram Gradient Boosting	Superconducting → Semiconductor	−0.39 ± 0.0099
Histogram Gradient Boosting	Semiconductor → Superconducting	−433.60 ± 86.55

## Data Availability

The data presented in this study are openly available in the GitHub repository at https://github.com/quanfu2-c/entropy-ML-connectivity, accessed on 7 December 2025.
